# Comparative Outcomes of Superomedial and Inferior Pedicles in Breast Reduction and Mastopexy: A Meta-Analysis of 5123 Breasts

**DOI:** 10.1007/s00266-024-04389-0

**Published:** 2024-10-21

**Authors:** Yousef Tanas, Julie Tanas

**Affiliations:** Faculty of Medicine, Alexandria University, Alexandria, Egypt

**Keywords:** Breast reduction, Mastopexy, Superomedial pedicle, Inferior pedicle, Patient satisfaction, Complications

## Abstract

**Background:**

Superomedial and inferior pedicles are two commonly used techniques in breast reduction and mastopexy. This systematic review and meta-analysis aims to compare the clinical outcomes associated with these two techniques.

**Methods:**

PubMed, Scopus, and Web of Science were searched for relevant studies. We included all studies with data comparing superomedial and inferior pedicles. Statistical analyses were performed using RevMan version 5.4.

**Results:**

The search yielded 1075 studies, of which 15 were included in the meta-analysis, encompassing 2633 patients (5123 breasts), with 3491 breasts receiving superomedial pedicles and 1632 breasts receiving inferior pedicles. Superomedial pedicles were associated with significantly shorter operative length (MD = − 24.71, 95% CI = − 37.63 to − 11.79*, p* = 0.0002), higher BREAST-Q breast satisfaction scores (MD = 10.34, 95% CI = 7.72 to 12.96, *p* < 0.00001), lower infection rates (RR = 0.46, 95% CI = 0.24 to 0.86, *p* = 0.02), higher incidence of seroma (RR = 3.00, 95% CI = 1.15 to 7.79, *p* = 0.02), and higher incidence of decreased nipple–areola complex (NAC) sensation (RR = 1.50, 95% CI = 1.12 to 2.01, *p* = 0.006). No significant differences were observed in asymmetry, fat necrosis, NAC loss, and hematoma.

**Conclusion:**

Superomedial pedicles demonstrated higher incidences of decreased NAC sensation and seroma formation, lower incidence of infection, shorter operative length, and higher BREAST-Q breast satisfaction scores compared to inferior pedicles. Further research is needed to confirm these findings and explore the long-term aesthetic outcomes associated with both techniques.

**Level of Evidence III:**

This journal requires that authors assign a level of evidence to each article. For a full description of these Evidence-Based Medicine ratings, please refer to the Table of Contents or the online Instructions to Authors www.springer.com/00266.

**Supplementary Information:**

The online version contains supplementary material available at 10.1007/s00266-024-04389-0.

## Introduction

Breast reduction and mastopexy are essential surgical interventions designed to address both functional and aesthetic concerns associated with macromastia and breast ptosis. Women seeking these procedures often experience physical discomfort, such as back and neck pain, skin irritation, and limitations in physical activity, as well as psychological distress due to dissatisfaction with their breast appearance [1, 2]. The primary goal of these surgeries is to reduce breast volume, lift the breasts, and improve overall breast shape, thereby enhancing the patient’s quality of life [3–6].

The choice of surgical technique is paramount in achieving successful outcomes in breast reduction and mastopexy. Among the various techniques available, the superomedial and inferior pedicles are widely recognized for their effectiveness in maintaining nipple–areola complex (NAC) viability, achieving optimal breast aesthetics, and minimizing complications. Each technique has its unique approach and advantages, making the decision on which method to use a critical aspect of surgical planning.

First described in 1957 by Arie [7], the superior pedicle was initially considered unreliable for long pedicle reconstructions due to compromised nipple viability [8]. This technique was refined by Orlando and Guthrie in 1975 [9], incorporating more medial parenchyma into the pedicle to ensure adequate vascularity of the nipple–areolar complex. Subsequent studies using this approach have demonstrated its safety, even in larger breast reductions, with complication rates comparable to the inferior pedicle technique [10]. Additionally, studies have shown that the superomedial pedicle technique results in shorter operative times, better cosmetic durability (less bottoming out or pseudoptosis over time), and a superior aesthetic appearance with fuller medial breast volumes and enhanced cleavage [11]. In practice, the superomedial pedicle breast reduction is typically performed using a Wise-pattern skin incision. The procedure begins with the de-epithelialization of the pedicle, followed by the en bloc resection of breast skin and parenchyma. The superomedial pedicle is then separated from the lateral breast pillar to allow for its rotation and final inset [12]. Studies, including those by Hall-Findlay and Shestak [13], have shown that the superomedial pedicle has a robust blood supply primarily from the second and third intercostal spaces of the internal mammary artery. This vascular reliability makes it comparable to other pedicles in terms of safety and complication rates. For example, a large retrospective review of 938 superomedial reduction mammaplasties reported an overall low complication rate, with partial nipple necrosis occurring in only 3% of cases [14]. This rate is comparable to or even lower than some reports for the inferior pedicle technique

Conversely, the inferior pedicle technique, popularized by Robbins in 1977, involves preserving a tissue flap from the lower part of the breast. This technique has gained widespread acceptance due to its straightforward approach and reliability in maintaining NAC viability. The inferior pedicle is particularly effective in patients with significant breast hypertrophy, as it allows for substantial tissue resection while maintaining a natural breast shape. Traditional teachings have favored the inferior pedicle technique due to its perceived vascular reliability, making it the preferred method for the majority (69%) of plastic surgeons in the USA [15]. Nonetheless, the superomedial pedicle technique is gaining acceptance as a reliable vascular pedicle and an attractive alternative for reduction mammaplasty.

Despite the proven efficacy of both techniques, the literature reveals a lack of consensus regarding their comparative advantages and disadvantages. Various studies have reported different outcomes concerning complication rates, operative times, and patient satisfaction, leading to ongoing debate among surgeons. Some studies suggest that the superomedial pedicle may offer superior aesthetic outcomes, while others advocate for the reliability and simplicity of the inferior pedicle.

Given these uncertainties, a systematic review and meta-analysis are necessary to provide a comprehensive evaluation of the clinical outcomes associated with the superomedial and inferior pedicle techniques. By synthesizing data from multiple studies, this analysis aims to identify which technique offers better results in terms of complication rates, operative length, and patient-reported outcomes, such as BREAST-Q satisfaction scores. By addressing these objectives, this study aims to provide evidence-based recommendations that can guide surgeons in selecting the most appropriate technique for breast reduction and mastopexy.

## Methods

The protocol for this paper was registered on PROSPERO (CRD42024542937), and the regulations of the preferred reporting items of systematic reviews and meta-analysis (PRISMA) were followed [16].

### Search Strategy

A literature search of PubMed, Scopus, and Web of Science was conducted on May 22, 2024, using key terms such as ((Superomedial) OR (Superior)) AND (Inferior) AND (Pedicle) AND (Breast) to identify relevant studies.

### Inclusion and Exclusion Criteria

These criteria aim to ensure the inclusion of relevant studies while excluding those that do not meet the scope or quality standards of the analysis.

### Inclusion Criteria

This meta-analysis encompassed observational studies written in English that included case-control, cohort, and cross-sectional studies involving adult patients (≥ 18 years) undergoing breast reduction or mastopexy with either superomedial or inferior pedicles and providing information on the incidence of complications and BREAST-Q breast satisfaction.

### Exclusion Criteria

Commentary, reviews, systematic reviews, meta-analyses, case reports, case series, or studies involving animal research were not included. Upon encountering duplicate studies, we included the most recent studies with the greatest number of participants.

### Study Selection

Two independent reviewers evaluated the studies based on our criteria. If a consensus cannot be reached, a volunteering plastic surgeon was consulted to settle the conflict.

### Data Extraction and Quality Assessment

Two reviewers independently extracted the data from each study. To ensure accuracy, the data were then compared. If a consensus cannot be reached, a volunteering plastic surgeon was consulted to settle the conflict. The following details were extracted from the eligible studies to create the baseline and summary data: first author’s last name, year the study was published, the study design, number of patients, number of breasts, age, BMI, number of smokers, number of patients with diabetes mellitus, follow-up time, the amount of resected breast tissue, Wise or vertical scar, and preoperative NAC to sternal notch distance. For the outcomes data, the following information was extracted: BREAST-Q breast satisfaction, hematoma, infection, nipple–areola loss/necrosis, loss/decreased sensation to nipple/breast, fat necrosis, hypertrophic scarring, wound dehiscence, asymmetry, operative length. The quality of the included articles was assessed according to the Newcastle–Ottawa scale (NOS), where a score of 7 or more was considered a high-quality paper. In contrast, a score of 6 or less was deemed low [17].

### Data Analysis

The analysis was performed using Review Manager version 5.4. Continuous data were represented as mean differences (MD), and dichotomous data were represented as risk ratios (RR), each with their respective 95% confidence intervals (CI). A fixed-effect model was applied when the data exhibited insignificant heterogeneity. In contrast, a random-effect model was employed in cases of significant heterogeneity (*p* < 0.05). To address heterogeneity, a leave-one-out test was utilized. Results were considered significant at a *p* value < 0.05.

### Definition of Heterogeneity

It is the variation or diversity in outcomes among the studies included in the meta-analysis. It may be due to several factors, such as the characteristics of the participants, study designs, the methods of analysis, or other sources of bias [18].

## Results

### Summary of Studies

**Fig. 1:**
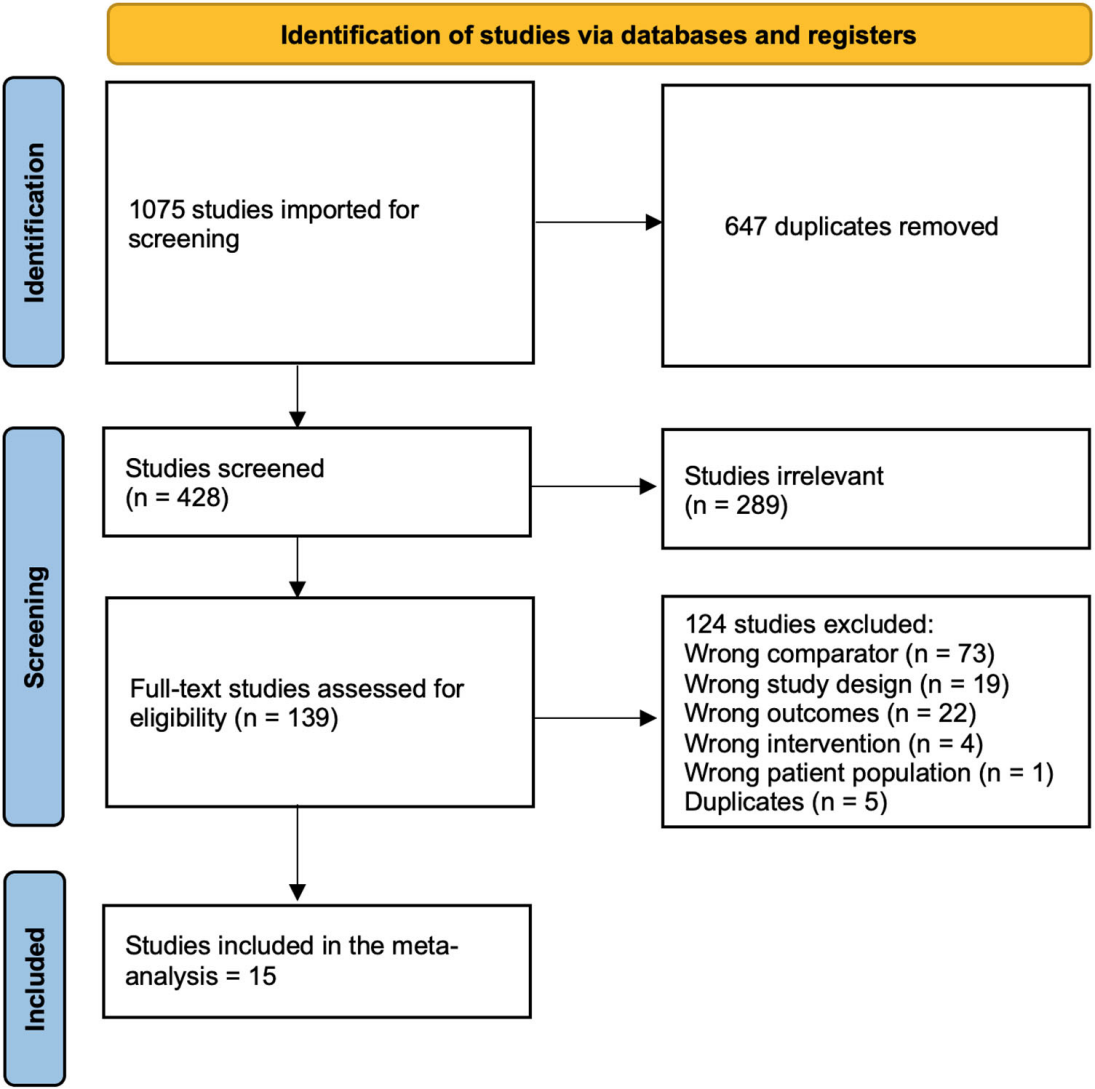
PRISMA flow diagram

After a comprehensive search of the literature, 1075 studies were found and 428 were eligible for title and abstract screening after removal of 647 duplicates. Of the 428, 289 were irrelevant and 139 studies were eligible for full-text screening. Finally, 15[14, 19–32] studies were included in the meta-analysis after full-text screening, as shown in the PRISMA diagram [16] in Fig.1.

The overall quality of the included studies was good in 10 studies and fair in 4 studies, as shown in Supplementary Table 1.

The total number of patients included in the study is 2633 (5123 breasts), 1795 patients (3491 breasts) in the superomedial pedicle group, and 838 patients (1632 breasts) in the inferior pedicle group.

In the superomedial pedicle group, 2535 breasts had a Wise incision, and 570 breasts were vertical incision. In the inferior pedicle group, 1460 breasts were Wise incision, and only 18 breasts were vertical incision. The remaining breasts in both pedicle groups were not specified as to whether they were Wise or vertical incisions.

Other baseline data are shown in Table 1.

**Table 1:** Baseline characteristics of included studies

Study Author, year	Number of patients		Number of breasts		
	SMP	IP	SMP	IP	SMP
Sandsmark [19]	228	26	456	52	30.4
Antony [20]	50	50	100	100	31.4
Ogunleye [21]	90	34	178	68	39.98
Schulz [22]	11	72	18	139	43.5
Kemaloglu [23]	25	25	50	50	38.8
Kame [24]	10	9	20	18	30.6
Jorgensen [25]	323	34	646	68	40.31
Sapino [26]	36	22	72	44	33.9
Toplu [14]	99	32	198	64	46
Watfa [27]	42	31	84	62	33.6
Palve [28]	477	201	865	363	50
Payton [29]	127	147	254	294	
Turan [30]	96	45	188	90	48.51
Cunning [31]	60	41	120	82	38
Ercan [32]	121	69	242	138	Geriatric:68.1 non-geriatric:39.6

Age (years)	BMI		Smoker		Diabetes Mellitus		Follow-up time (months)	
IP	SMP	IP	SMP	IP	SMP	IP	SMP	IP
30.4	NA	NA	NA	NA	NA	NA	NA	NA
31.6	30.8	31.8	NA	NA	NA	NA	12	12
37.24	29.82	32.21	3	1	2	6	12	12
43.5	NA	NA	NA	NA	NA	NA	6	6
41.5	32.2	31.5	1	1	3	2	19.6	19.6
30.11	36.62	33.08	NA	NA	NA	NA	6	6
39.65	24.69	25.14	49	7	NA	NA	74.16	73.2
38.4	29	28.4	7	4	NA	NA	24	24
36.5	29		8	0	6	0	48	48
38.8	28.6	28.5	9	8	NA	NA	24	24
52	26.8	28	26	13	25	8	27.6	38.4
35.57	30.16		NA	NA	11		4.5	
48.82	NA	NA	NA	NA	NA	NA	NA	NA
38	17–29.9: 23 30–39.9: 26 ≥40: 0	17–29.9: 23 30–39.9: 13 ≥40: 5	NA	NA	NA	1	1.5-4	1.5-4
Geriatric:67.4 non-Geriatric:43.2	Geriatric:28.4 non-geriatric: 28.3	Geriatric: 28.1 non-geriatric: 29	15	20	7	8	32.4	

Amount of resected breast tissue (g)		Wise (inverted T) or Vertical scar		Preoperative NAC to sternal notch distance (cm)	
SMP	IP	SMP	IP	SMP	IP
R: 547; L: 542		Wise: 456 breasts	Wise: 52 breasts	NA	NA
815.2	839.8	vertical: 100 breasts	Wise: 100 breasts	NA	NA
546.02	1090.47	vertical: 178 breasts	Wise: 68 breasts	NA	NA
459.07	769.2	vertical: 18 breasts	Wise: 139 breasts	NA	NA
R:1320; L:1355	R:1380; L:1310	Wise: 50 breasts	Wise: 50 breasts	30	30
NA	NA	Vertical: 20 breasts	Vertical: 18 breasts	NA	NA
967.85	959.81	Wise: 646 breasts	Wise: 68 breasts	31.6	33.4
698.9 per breast	602.1 per breast	Wise: 72 breasts	Wise: 44 breasts	31.4	31.2
R:100600; L:1090	R:1100; L:1100	Wise: 171 patients vertical: 15 patients		R&L:31	R&L:30
672.2	631.3	Wise: 84 breasts	Wise: 62 breasts	NA	NA
R:479; L:480	R:491; L:500	Wise: 865 breasts	Wise: 363 breasts	R&L:30.5	R:30.5 L:31
R: 596.21; L: 613.64		Vertical: 254 breasts	Wise: 294 breasts	NA	NA
R:870 L:815	R:820; L:800	NA	NA	NA	NA
805.8	668.5	Wise: 120 breasts	Wise: 82 breasts	33	33.1
Geriatric:583 non-geriatric:594	Geriatric:602 non-geriatric: 659	Wise: 242	Wise: 138	NA	NA

*SMP* superomedial pedicle; *IP* inferior pedicle; *BMI* body mass index; *NA* not available; *R* right breast; *L* left breast; and *NAC* nipple–areola complex

### Outcomes

#### Asymmetry

**Fig. 2:**
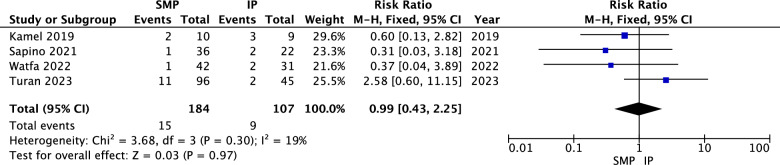
Asymmetry forest plot

The pooled analysis indicated no statistically significant difference between superomedial and inferior pedicles regarding asymmetry (RR = 0.99, 95% CI = 0.43 to 2.25, *p* = 0.97). Heterogeneity was not significant (*P* = 0.30, I^2^ = 19%) as shown in Fig. 2.

#### Wound Dehiscence

**Fig. 3:**
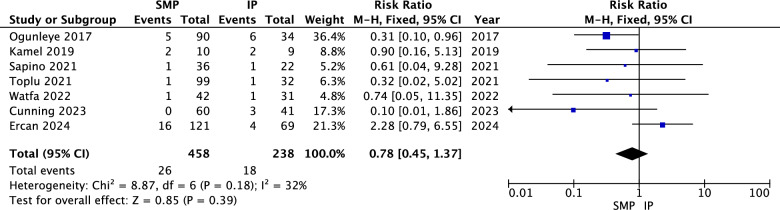
Wound dehiscence forest plot

The pooled analysis showed no statistically significant difference between superomedial and inferior pedicles in terms of wound dehiscence (RR = 0.78, 95% CI = 0.45 to 1.37, *p* = 0.39). Heterogeneity was not significant (*P* = 0.18, I^2^ = 32%) as shown in Fig. 3.

#### Hypertrophic Scarring

**Fig. 4:**
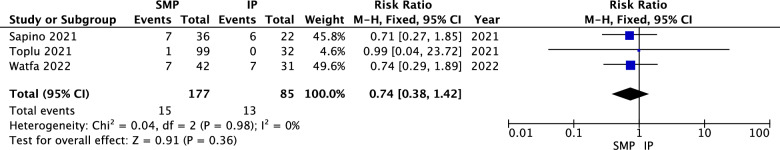
Hypertrophic scarring forest plot

The pooled analysis indicated no statistically significant difference between superomedial and inferior pedicles regarding hypertrophic scarring (RR = 0.74, 95% CI = 0.38 to 1.42, *p* = 0.36). Heterogeneity was not significant (*P* = 0.98, I^2^ = 0%) as illustrated in Fig. 4.

#### Fat Necrosis

**Fig. 5:**
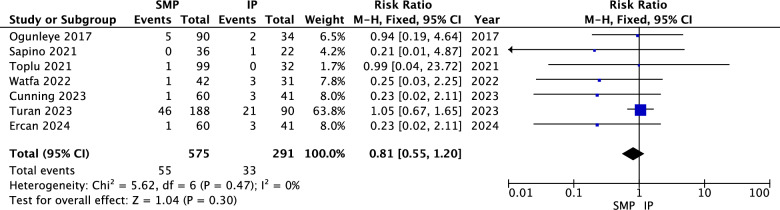
Fat necrosis forest plot

The analysis revealed no statistically significant difference between superomedial and inferior pedicles in terms of fat necrosis (RR = 0.81, 95% CI = 0.55 to 1.20, *p* = 0.30). Heterogeneity was not significant (*P* = 0.47, I^2^ = 0%) as shown in Fig. 5.

#### NAC Decreased Sensation

**Fig. 6:**
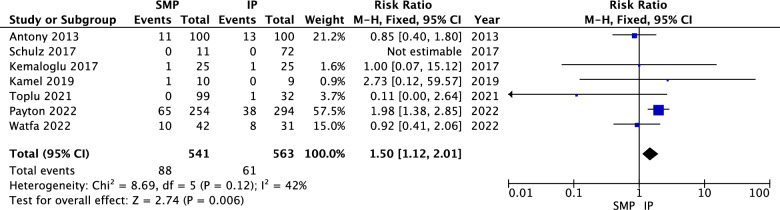
NAC decreased sensation forest plot

The pooled analysis showed a statistically significant association between superomedial pedicle and a higher incidence of decreased NAC sensation compared to inferior pedicle (RR = 1.50, 95% CI = 1.12 to 2.01, *p* = 0.006). Heterogeneity was not significant (*P* = 0.12, I^2^ = 42%) as shown in Fig. 6.

#### NAC Loss or Necrosis

**Fig. 7:**
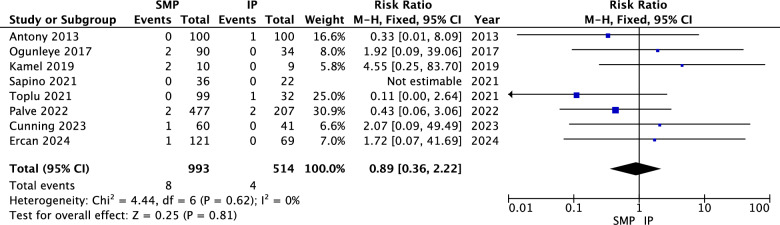
NAC loss forest plot

The analysis indicated no statistically significant difference between superomedial and inferior pedicles regarding NAC loss (RR = 0.89, 95% CI = 0.36 to 2.22, *p* = 0.81). Heterogeneity was not significant (*P* = 0.62, I^2^ = 0%) as shown in Fig. 7.

#### Infection

**Fig. 8:**
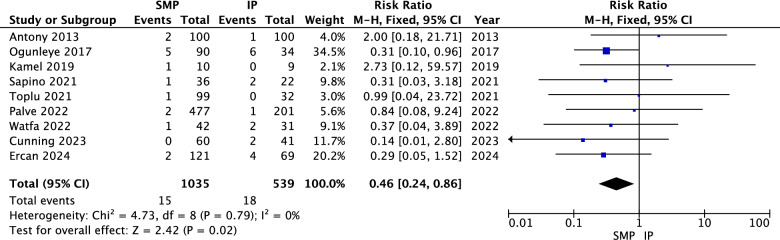
Infection forest plot

The pooled analysis revealed a statistically significant association between superomedial pedicle and lower infection rates compared to inferior pedicle (RR = 0.46, 95% CI = 0.24 to 0.86, *p* = 0.02). Heterogeneity was not significant (*P* = 0.79, I^2^ = 0%) as shown in Fig. 8.

#### Hematoma

**Fig. 9:**
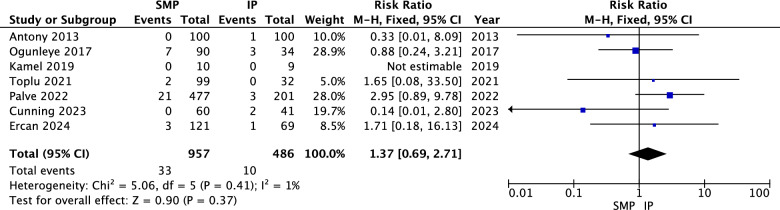
Hematoma forest plot

The analysis showed no statistically significant difference between superomedial and inferior pedicles regarding hematoma rates (RR = 1.37, 95% CI = 0.69 to 2.71, *p* = 0.37). Heterogeneity was not significant (*P* = 0.41, I^2^ = 1%) as illustrated in Fig. 9.

#### Seroma

**Fig. 10:**
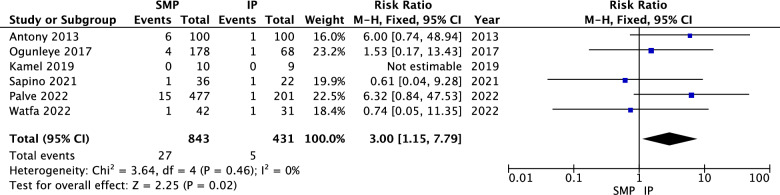
Seroma forest plot

The pooled analysis indicated a statistically significant association between superomedial pedicles and increased incidence of seroma compared to inferior pedicles (RR = 3.00, 95% CI = 1.15 to 7.79, *p* = 0.02). Heterogeneity was not significant (*P* = 0.46, I^2^ = 0%) as shown in Fig. 10.

#### Operative Length

**Fig. 11:**
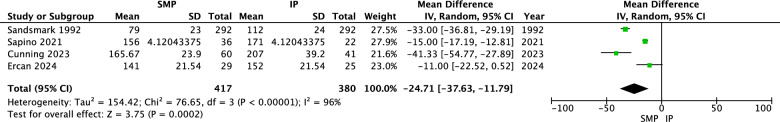
Operative length forest plot

The pooled analysis showed a statistically significant association between superomedial pedicles and a shorter operative length compared to inferior pedicles (MD = -24.71, 95% CI = -37.63 to -11.79, *p* = 0.0002). We observed significant heterogeneity among studies (*P* < 0.00001, I^2^ = 96%), which was not resolved by the leave-one-out test as shown in Fig. 11.

#### BREAST-Q Breast Satisfaction

**Fig. 12:**
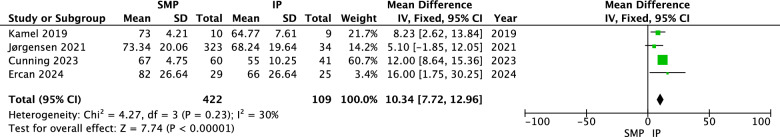
BREAST-Q breast satisfaction forest plot

The pooled analysis showed a statistically significant association between superomedial pedicles and a higher BREAST-Q breast satisfaction compared to inferior pedicles (MD = 10.34, 95% CI = 7.72 to 12.96, *p* < 0.00001). Heterogeneity was not significant (*P* = 0.23, I^2^ = 30%) as shown in Fig. 12.

#### BREAST-Q Satisfaction with Outcome

**Fig. 13:**
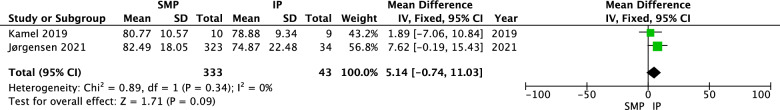
BREAST-Q satisfaction with outcome forest plot

The analysis showed no statistically significant difference between superomedial and inferior pedicles regarding BREAST-Q Satisfaction with outcome (RR = 5.14, 95% CI = 0.74 to 11.03, *p* = 0.09). Heterogeneity was not significant (*P* = 0.34, I^2^ = 0%) as illustrated in Fig. 13.

## Discussion

This meta-analysis compares the outcomes of superomedial versus inferior pedicle techniques in breast reduction and mastopexy. The analysis reveals notable differences in certain clinical outcomes, highlighting the strengths and weaknesses of each technique. The incidence of asymmetry, wound dehiscence, hypertrophic scarring, fat necrosis, NAC loss, and hematoma did not show statistically significant differences between the two techniques. This suggests that both superomedial and inferior pedicles provide similar outcomes in these specific complications, making either technique a viable option depending on the surgeon’s preference and patient-specific factors.

Although the superomedial pedicle demonstrated significant advantages in some critical areas (lower incidence of infection, shorter operative length, and higher BREAST-Q breast satisfaction), it was associated with higher incidences of seroma formation and decreased NAC sensation compared to the inferior pedicle. The potential for reduced NAC sensation in the superomedial pedicle compared to the inferior pedicle can be attributed to several anatomical and surgical factors. Hall-Findlay and Shestak (2015) (13) elucidated the detailed anatomy of the breast’s nerve supply, highlighting that NAC sensation primarily originates from the anterolateral branch of the 4th intercostal nerve. This nerve sends superficial and deep branches into the subcutaneous tissue and over the pectoralis fascia, respectively. In most reduction mammoplasty procedures, the superficial branch is often severed, except when a lateral pedicle is used. The deep branch can be preserved in both the superomedial and inferior pedicle techniques if the pectoralis fascia remains intact. However, the superomedial pedicle technique involves more extensive dissection and manipulation of the medial breast tissue, which can increase the risk of nerve damage. The mobilization of the breast tissue in the superomedial approach can disrupt the anterolateral branch of the 4th intercostal nerve more significantly than the inferior pedicle technique.

The findings by the largest included study for the NAC sensation outcome, Payton et al. [29], indicate that the patients were more likely to experience decreased sensation with a vertical skin resection, superomedial pedicle compared to Wise-pattern, inferior pedicle reduction. Interestingly, they noted that the only factor found to correlate with increased NAC sensation was longer operative times, and that for every 30 minute increase in operative duration, the odds of heightened sensation increased by 59%. This is a notable finding as the operative length was significantly shorter for the superomedial pedicle technique in our analysis. This finding is consistent with previous studies suggesting that the superomedial pedicle may have a higher incidence of NAC sensation loss but allows for more efficient surgical procedures, potentially reducing overall operating room time and associated costs [19, 26, 31–33] for several reasons. It requires less de-epithelialization due to the smaller surface area of the pedicle. The total distance of the pedicle border that needs to be isolated from surrounding tissue is also reduced. There is no need for substantial thinning or creation of flaps, and the resection can be performed in a single en bloc piece. These streamlined steps contribute to shorter operative times [33] but this could potentially be at the cost of an increased risk of NAC sensation loss and so careful surgical planning is imperative. Conversely, Courtiss and Goldwyn [34] reported a 35 percent rate of nipple numbness two years after an inverted T, inferior pedicle breast reduction. This substantial rate of nipple numbness highlights the long-term sensory complications that can arise with the inferior pedicle technique, despite its perceived vascular reliability and straightforward approach. The differences in sensory outcomes between the techniques may be influenced by the extent of tissue handling and the preservation of nerve pathways during surgery. These contradicting findings emphasize the complexity of sensory outcomes in breast reduction surgeries and underscore the need for individualized surgical planning. Surgeons must carefully consider the specific anatomical and procedural factors that influence NAC sensation when choosing the appropriate technique. The inferior pedicle technique, while widely accepted and preferred by many plastic surgeons due to its straightforward approach and reliability, did not show superiority in most outcomes measured in this meta-analysis except for the lower incidences of seroma formation and decreased NAC sensation, which are both important complications to consider, notwithstanding. The traditional preference for the inferior pedicle is often based on its perceived vascular reliability, particularly in patients with significant breast hypertrophy. Critics of the superomedial pedicle argue that the superomedial pedicle is less effective for very large reductions due to the amount of parenchyma retained within the pedicle. While this may be true for vertical pattern reductions, the Wise pattern allows for substantial reduction with en bloc resection of the inferior breast tissue, as well as thinning of the lateral flap and distal pedicle if necessary. In a matched cohort study included in our analysis, Antony et al. [20] found no significant differences in complication rates between large reductions (defined as more than 1000 g per breast) and smaller reductions, concluding that the superomedial pedicle can be effectively used for reductions exceeding 1000 g per breast. A series by Brown et al. [33] also included numerous patients with reductions over 1000 g, with similarly low complication rates. These findings are in line with our analysis which suggests that the superomedial pedicle can offer comparable, if not superior, outcomes in several important metrics even with large reductions.

Nonetheless, the analysis of seroma rates indicated a statistically significant higher incidence with the superomedial pedicle, especially in the study by Palve et al. [28] which showed an RR of 6.32. The increased incidence of seroma formation can be attributed to several anatomical and procedural factors. The superomedial pedicle technique involves more extensive dissection and mobilization of the medial and superior breast tissue. This increased tissue handling can disrupt the lymphatic channels, leading to fluid accumulation and seroma formation. Additionally, the larger surface area of tissue that is manipulated and repositioned in the superomedial pedicle technique can contribute to a higher risk of seroma. The creation of a larger surgical pocket and potential dead space within the breast can also facilitate fluid accumulation. In contrast, the inferior pedicle technique, with its more straightforward dissection and preservation of a greater amount of surrounding tissue, may lead to less disruption of the lymphatic system and a reduced likelihood of seroma. These anatomical considerations highlight the importance of meticulous surgical technique and careful postoperative management to minimize the risk of seroma in patients undergoing superomedial pedicle breast reduction.

The superomedial pedicle technique is known for providing enhanced medial fullness and superior cleavage due to the incorporation of more medial parenchyma into the pedicle, supporting better upper pole projection and a more youthful breast contour [24]. It also has a reduced tendency for bottoming out or pseudoptosis over time as shown in the included study by Kamel et al. [24], attributed to the robust support provided by the medial pedicle, leading to longer-lasting aesthetic results. Although we did not perform analysis for this outcome as Kamel et al. was the only study reporting the incidence for bottoming out, our meta-analysis shows that the BREAST-Q breast satisfaction scores were also significantly higher for the superomedial pedicle technique. This potentially reflects the overall better aesthetic outcomes and satisfaction associated with this technique. These findings are also similar to the study by Makboul et al. (2017) [11] which was not included in our analysis as BREAST-Q was not used and a non-standardized questionnaire was used instead. They conducted a long-term follow-up (6 years) comparing patient satisfaction after reduction mammaplasty using superomedial versus inferior pedicles. They found that patients with the superomedial pedicle reported higher satisfaction with breast projection, contour, and overall outcomes, with significantly less recurrence of ptosis compared to the inferior pedicle. The study also noted better scar acceptance and long-term aesthetics with the superomedial technique.

In another study by Zhu et al. (2016) [35] not included in our analysis, they utilized three-dimensional breast imaging to quantify and compare the postoperative volumetric and morphologic outcomes between inferior and superomedial pedicle breast reductions. In their study, reduction mammaplasty was performed using either a superomedial pedicle with a modified Robertson skin incision or an inferior pedicle with a Wise-pattern skin incision. Patients were matched based on postoperative breast size, BMI, and age, with postoperative three-dimensional photographs taken at 1 to 3 months and 6 to 12 months. The study included 13 patients (26 breasts) with superomedial pedicles and 14 patients (28 breasts) with inferior pedicles. They found that the average weight resected was significantly lower in the superomedial cohort (417 cc vs. 846 cc, *p* < 0.01). At 1 to 3 months postoperatively, significant differences were observed in sternal notch-to-nipple distance and superior pole fullness, with the superomedial pedicle showing better outcomes. This difference in sternal notch-to-nipple distance persisted at 6 to 12 months, while superior pole fullness equalized between the groups. Interestingly, the superomedial pedicle cohort exhibited less medial pole fullness and less increase in areola surface area over time compared to the inferior pedicle cohort.

Our meta-analysis has several strengths. Firstly, it includes a large sample size of 2633 patients and 5123 breasts, providing robust statistical power to detect differences in outcomes between the superomedial and inferior pedicle techniques. The comprehensive literature search and inclusion of multiple high-quality studies enhance the reliability of the findings. By focusing on a range of outcomes, including complication rates, operative length, and patient-reported satisfaction through BREAST-Q scores, this study offers a thorough comparison of the two techniques. Another strength is the rigorous methodology employed in this meta-analysis. The use of both fixed and random-effects models, depending on the presence of heterogeneity, ensures appropriate statistical analysis. Additionally, the study follows the PRISMA guidelines for systematic reviews and meta-analyses, ensuring transparency and reproducibility of the results.

Despite its strengths, this study has several limitations. The heterogeneity among the included studies may be a concern, particularly for the operative length outcome, which could not be solved by leave-one-out sensitivity test, though all the included studies have shown that the superomedial technique has consistently shorter operative time compared to the inferior pedicle technique. Further, the meta-analysis relies on published studies, which may introduce publication bias. Studies with negative or non-significant findings are less likely to be published, potentially skewing the overall results. Additionally, the included studies vary in their design, patient populations, and reporting methods, which can introduce variability and affect the comparability of the results. Another limitation is the retrospective nature of many included studies, which are prone to bias and confounding factors. Prospective, randomized controlled trials are needed to confirm these findings and provide higher-quality evidence. The analysis also does not account for long-term outcomes (e.g., bottoming out or pseudoptosis), as most included studies have limited follow-up periods and do not report the incidences of this outcome. Long-term data are essential to assess the durability of the results and the occurrence of late complications. Also, a significant limitation in our analysis is the lack of subgroup analyses comparing outcomes with Wise and vertical incisions. This was due to the limited number of included studies examining the vertical incision, particularly in the inferior pedicle group, which had only 18 breasts (one study (24)) with a vertical incision. Finally, while the BREAST-Q scores provide valuable insights into patient satisfaction, they are subjective measures and may be influenced by individual patient expectations and experiences. Objective measures of aesthetic outcomes and quality of life would complement these findings and provide a more comprehensive assessment of the techniques.

## Conclusion

This meta-analysis provides a comprehensive comparison of the superomedial and inferior pedicle techniques in breast reduction and mastopexy. Both techniques demonstrated similar outcomes in terms of asymmetry, wound dehiscence, hypertrophic scarring, fat necrosis, NAC loss, and hematoma. The superomedial pedicle was associated with shorter operative times, higher BREAST-Q breast satisfaction scores, and lower infection rates, but also showed higher incidences of seroma formation and decreased NAC sensation. These findings suggest that while the superomedial pedicle offers certain advantages in operative efficiency and patient satisfaction, it also carries specific risks that need careful consideration. Further high-quality, prospective research is necessary to confirm these results and to explore the long-term aesthetic outcomes associated with both techniques. By providing a balanced assessment of both methods, this study aims to guide surgeons in selecting the most appropriate technique based on individual patient factors and surgical goals.

## Supplementary Information

Below is the link to the electronic supplementary material.

Supplementary file1 (XLSX 17 KB)
